# Supplementary material for: SMOC1 colocalizes with Alzheimer’s disease neuropathology and delays Aβ aggregation
Source: Res Sq. 2024 Nov 1:rs.3.rs-5229472. Preprint. [Version 1] doi: 10.21203/rs.3.rs-5229472/v1 (PMC11581049; doi:10.21203/rs.3.rs-5229472/v1)
Supplement: Supplement 1 [file NIHPPRS5229472V1-supplement-1.pdf]

Supplementary Files

This is a list of supplementary files associated with this preprint. Click to download.

- SuppFig1.tiff
- SuppFig2.tif
- SuppFig3.png
- SuppFig4.tiff
